# Bilateral congenital choanal atresia and osteoma of ethmoid sinus with supernumerary nostril: a case report and review of the literature

**DOI:** 10.1186/1752-1947-5-583

**Published:** 2011-12-20

**Authors:** Xue-zhong Li, Xiao-lan Cai, Lei Zhang, Xue-feng Han, Xiao Wei

**Affiliations:** 1 Department of Otolaryngology, Qilu Hospital, Shandong University, Ji'nan 250012, China

## Abstract

**Introduction:**

Congenital choanal atresia is a relatively rare deformity, especially bilateral congenital choanal atresia. We report a case of bilateral congenital choanal atresia in a 22-year-old Chinese man, who was also diagnosed with congenital right accessory nasal deformity, osteoma of his left ethmoid sinus and congenital keratoleukoma of his right eye.

**Case presentation:**

A 22-year-old Chinese man presented with mouth breathing, sleep snoring and difficult feeding after birth, with no olfactory sensation. Three-dimensional computed tomography revealed bilateral choanal atresia and a high density bony shadow in his left ethmoid sinus that extended to his left frontal sinus.

**Conclusions:**

Choanal atresia is often accompanied by other congenital abnormalities. To the best of our knowledge, this is the first report of choanal atresia accompanied by congenital accessory nasal deformity and congenital keratoleukoma.

## Background

Choanal atresia was first reported by Johann Roderer in 1755 [1]. Its incidence is about 8.2 per 100, 000 [2] and the primary symptoms, such as bilateral imperforation, dyspnea and inability to suck after birth, are attributed to postnatal nasal obstruction. In serious cases, ensuing suffocation can lead to death. We present a rare case of bilateral congenital choanal atresia, which was accompanied by congenital right accessory nasal deformity, osteoma of his ethmoid sinus, and congenital keratoleukoma of his right eye.

## Case presentation

A 22-year-old Chinese man presented with mouth breathing, sleep snoring, and loss of olfactory sensation. Difficult feeding after birth was reported, without dyspnea or asphysia. He had no relevant family history.

**Figure 1:**
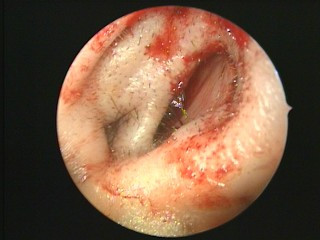
**Congenital right accessory nasal deformity**. The right anterior naris was divided into two parts by a barrier diaphragm protruding from a lateral wall.

Physical examination revealed severe closed rhinolalia and no airflow in both of his anterior naris. Three nostrils were identified on his external naris. His left naris, round-shaped and about 1 cm in diameter, was normal. However, his right anterior naris was divided into two parts by a barrier diaphragm which protruded from the lateral wall (Figure 1). The medial part of his right naris was relatively large, similar to the left, and connected to the nasal cavity, while the lateral part was approximately 0.3 cm in diameter and did not communicate with the nasal cavity.

**Figure 2:**
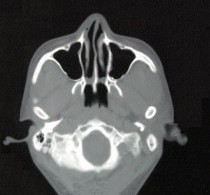
**Bilateral choanal atresia in axial C**. Atresia CT revealed bilateral choanal atresia.

**Figure 3:**
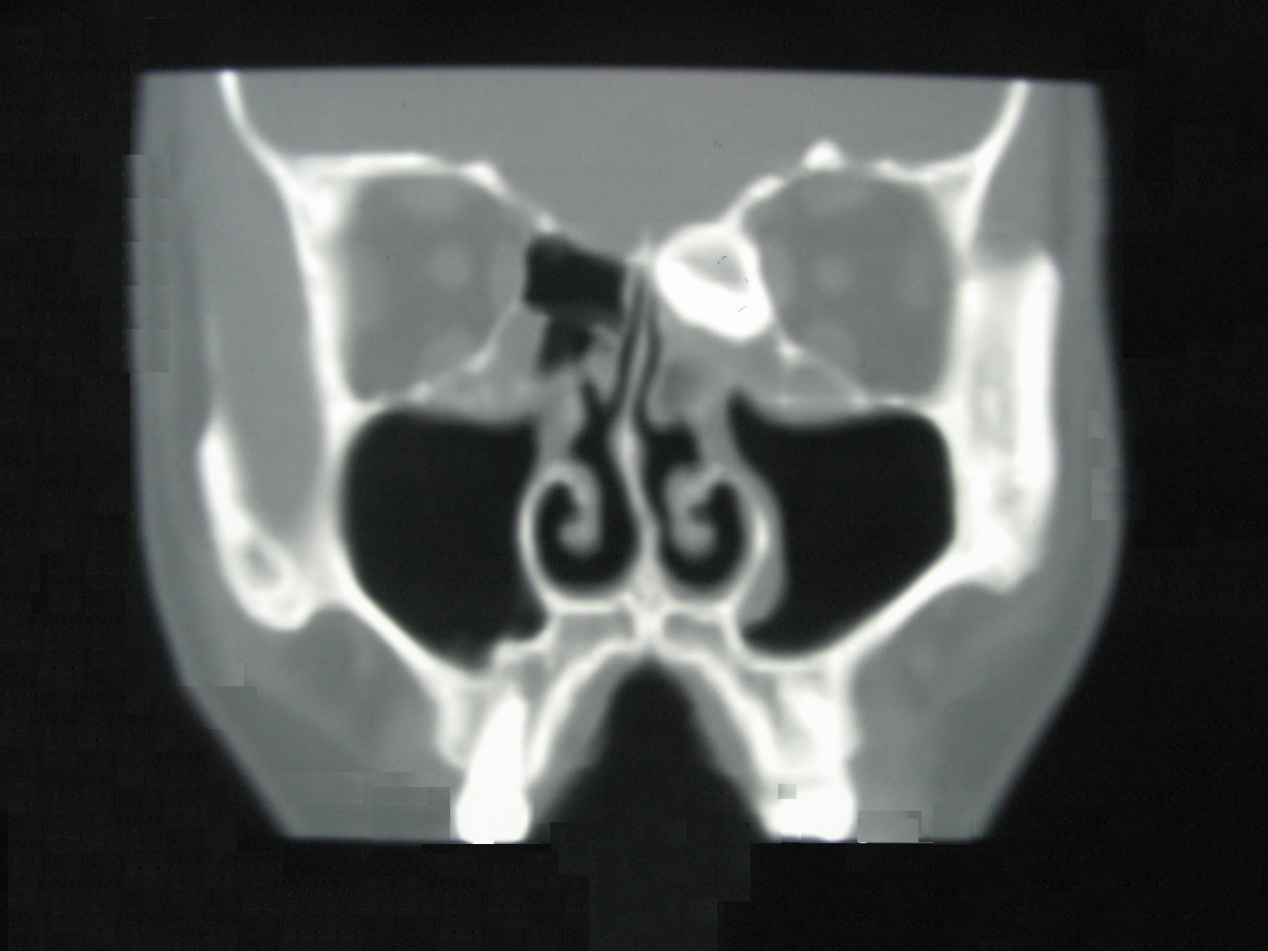
**Ethmoid osteoma**. CT revealed a high-density bony shadow in his left ethmoid sinus.

**Figure 4:**
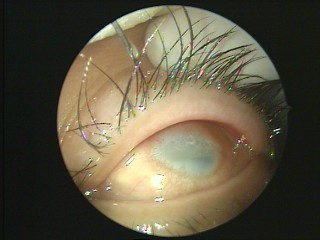
**Congenital keratoleukoma of his right eye**. The cornea of his right eye was covered by keratoleukoma.

Three-dimensional computed tomography (CT) revealed bilateral choanal atresia (Figure 2) and a high-density bony shadow in his left ethmoid sinus (Figure 3), which extended to the left frontal sinus. The cornea of his right eye was covered by keratoleukoma (Figure 4) without reflection to light, while his left eye was normal. His hearing and intelligence were normal.

**Figure 5:**
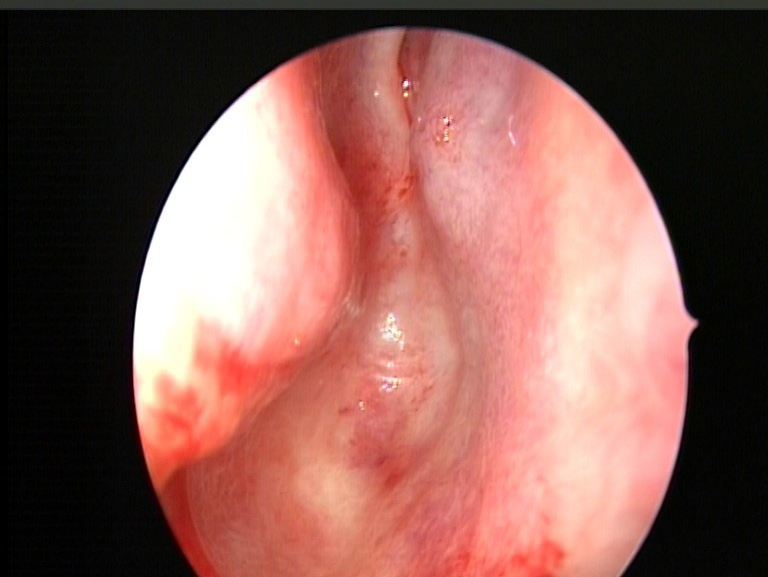
**Atresia of the right posterior naris under the nasal endoscopy**.

Our primary diagnosis was congenital right accessory nasal deformity (Figure 1), congenital bilateral atresia of the posterior nares (Figures 2 and 5), osteoma of the left ethmoid sinus (Figure 3) and congenital keratoleukoma of his right eye (Figure 4). He was operated on under general anesthesia in March 2007. The procedures included orthopedics of the right accessory nasal deformity, exploration and recanalization of the posterior nares, and resection of the osteoma in his left ethmoid sinus.

**Figure 6:**
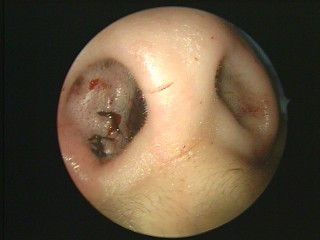
**The right anterior naris postoperation**. After the diaphragm was removed, the right anterior naris was recovered.

During the surgery, we found his right anterior naris was divided into two parts by a barrier diaphragm. The lateral part of the right anterior naris was a caecum and did not open to the nasal cavity. After the diaphragm was removed, the right anterior naris was recovered (Figure 6).

**Figure 7:**
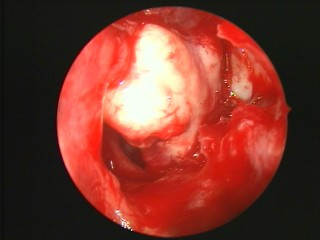
**Ethmoid osteoma**. A white bony mass in his left ethmoid sinus was identified clearly during the operation.

**Figure 8:**
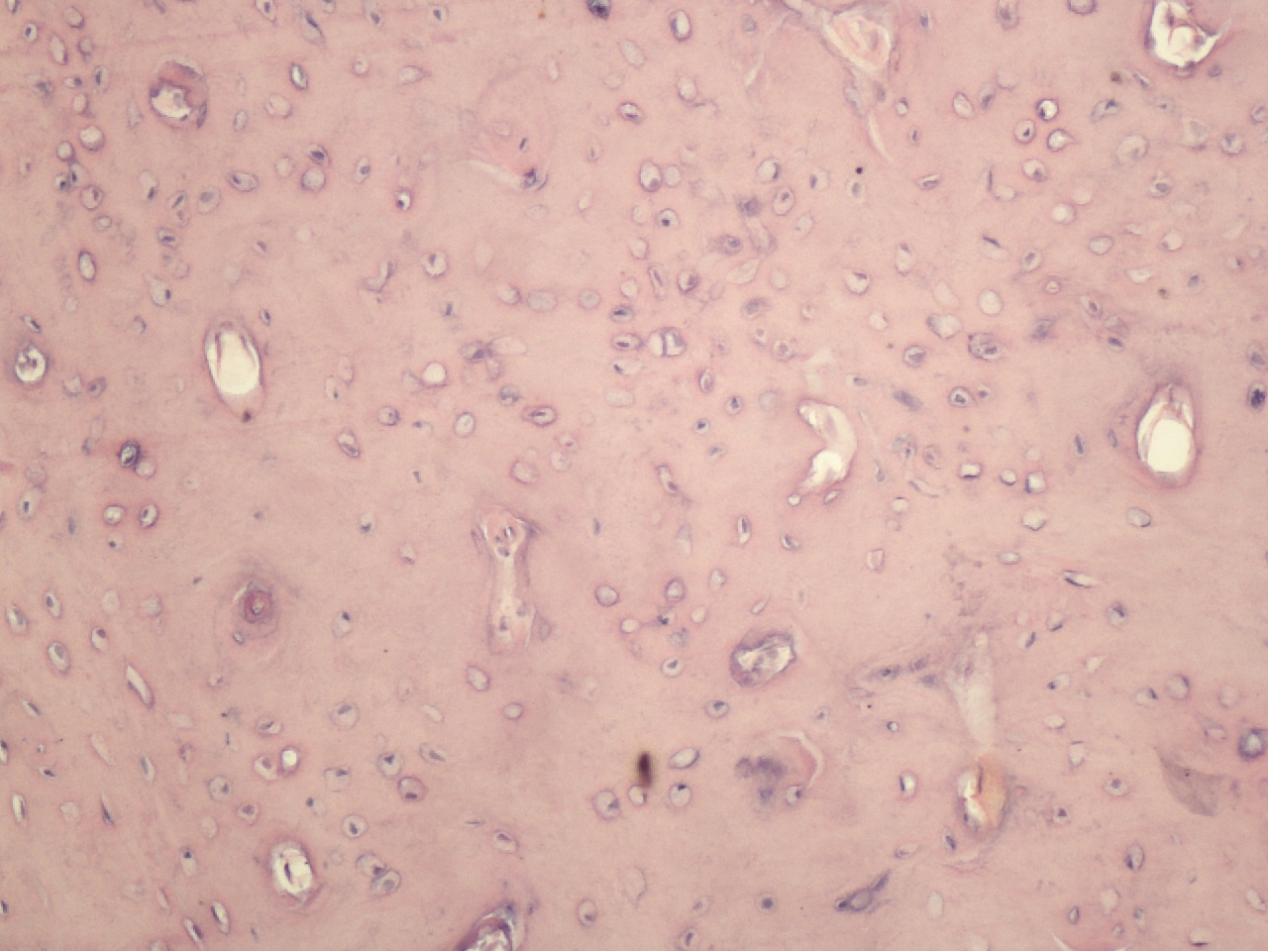
**Microphotograph of the tumor**. Hematoxylin and eosin stain (original magnification ×20).

Under nasal endoscopy, the uncinate process, ethmoid bulla and air cells were removed. The maxillary sinus ostium was opened to locate the plane of the lamina papyracea. Then, inside the ethmoid sinus, a white bony mass was identified clearly (Figure 7) and resected completely. The bony mass was irregular in shape, pale in color, and difficult to remove from the middle meatus. Its size was about 2.2 cm × 1.8 cm × 2.0 cm. The roof of the ethmoid and cribriform plate was examined carefully and found to be intact. Neither leakage of cerebrospinal fluid nor active bleeding occurred. Dense osteocarcinoma was diagnosed by postoperative pathology (Figure 8).

**Figure 9:**
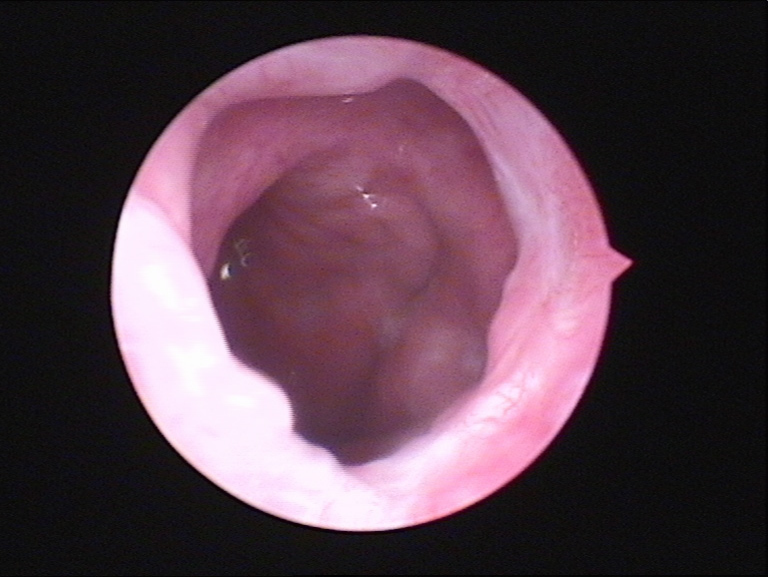
**Posterior naris postoperation**. The posterior nares were capacious on re-examine three months after the operation.

**Figure 10:**
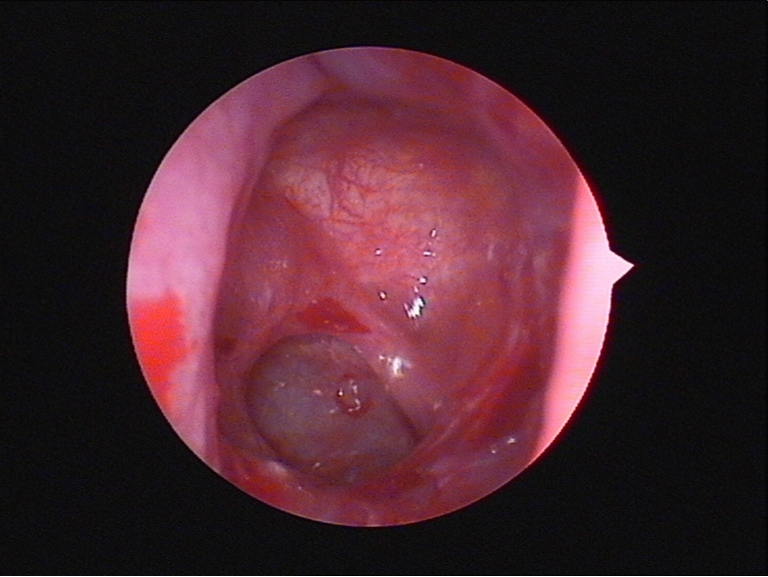
**Ethmoid sinus postoperation**. Re-examination three months after the operation.

A soft and elastic membrane was found to block both posterior nares. A cruciate incision was made in the membrane center and the openings were widened to the bony part of the posterior nares. A silica gel about 0.8 cm in diameter and 7 cm in length, with multiple holes in the lateral wall, was placed bilaterally from anterior to posterior nares to prevent re-stenosis. The silica gel was removed six weeks later. After more than three years of follow-up, both anterior and posterior nares were capacious and nasal ventilation was normal (Figures 9 and 10). Olfactory function recovered partially. Closed rhinolalia and sleep snoring were all remarkably relieved.

## Discussion

Congenital accessory nasal deformity is a supernumerary malformation in the external nose. Supernumerary malformation may appear in different ways. It may replace the normal structure or co-exist with it. Removal of the congenital accessory is the optimal treatment method. Tambwekar [3] reported one similar case. The patient in our report bore three anterior nares, however, this should be differentiated from three-nostril malformation, a type of cleft nose occurring when the median line of the nasal ridge presents as a major groove, or the mesenchymal tissue between two olfactory saccus fails to evolve into a thin but firm septal cartilage in the embryonic period, resulting in the nasal dorsum broadening and the cleft emerging. In our case, only the soft tissue formed the malformation in one side of the anterior nares, therefore this case was diagnosed as 'congenital accessory nasal deformity'.

Congenital atresia of the posterior nares can be categorized into two types: unilateral (60%) and bilateral (40%) atresia [4]. According to the closure tissue, it can be subdivided into membranous, bony or mixed bony-membranous atresia [5]. Although the incidence of congenital atresia of posterior nares is low, other congenital abnormalities often coexist [6]. In the case of our patient, congenital keratoleukoma of the right eye was an accompanying abnormality. Burrow *et al. *[7] stated that 26.4% of the choanal atresia they found was isolated, while 73.6% was associated with other anomalies. CHARGE syndrome may manifest with choanal atresia [8]. However, according to Verloes [9], our patient cannot be diagnosed as having CHARGE syndrome. Congenital bilateral atresia of the posterior nares is a very dangerous disease which can cause death in infants because the inability to nose breathe will lead to tremendous difficulty in sucking and feeding. Mouth breathing in the long term may result in malformation in the midface and/or the hard palate [10]. A high-arched palate was observed in our patient. In light of Brown's classification [5], our patient was diagnosed as having membranous congenital bilateral choanal atresia.

Osteoma is one common benign tumor which grows slowly in nasal sinuses. In most cases, patients do not suffer from any clinical symptoms; osteoma is found in imaging studies by accident. In general, osteoma of the paranasal sinuses occurs in the frontoethmoid region. The etiology is still unknown; however, there are three different hypothesis available suggesting that embryologic, traumatic or infective factors are involved [11]. The incidence of osteoma is 62% in the frontal sinus, 24% in the ethmoid sinus, and rarely in maxillary and sphenoid sinus [12]. An osteoma in nasal sinuses can be subcategorized into three basic histological types: compact, cancellous or mixed [13]. Imaging studies, especially CT and three-dimensional CT scans, are often applied for diagnoses [14]. If the osteoma does cause some discomfort, resection is recommended. The surgical approach depends on the location and size of the tumor. A small osteoma in the ethmoid sinus can be removed under nasal endoscope. For a large osteoma, lateral rhinotomy is preferred. When an osteoma infiltrates the front cranial basalis, a combined craniofacial approach is usually considered [15].

Keratoleukoma is a thin, opaque, abnormal coating on the cornea of the eye. It is one kind of corneal opacity. According to the thickness of the coating, a corneal opacity is graded into corneal nebula, corneal macula and keratoleukoma. The most common causes of corneal opacity are ocular trauma (50.6%), retinal disease (15.5%), measles (9.5%) and congenital etiologies (5.5%) [16]. Corneal opacities vary greatly in extent and location and are usually associated with other anomalies [17], for example the bilateral congenital choanal atresia and congenital right accessory nasal deformity in our patient. Because corneal opacity is a cause of blindness [18], timely treatment is crucial. A study has shown that 64.92% of patients underwent cosmetic treatment after the primary visit [16]. Corneal transplantation should be performed when needed [19].

## Conclusion

Congenital bilateral choanal atresia is a life-threatening disease in newborns. However, it can also be found in adults, who may present with mouth breathing, sleep snoring and symptoms of nasal obstruction. Choanal atresia is often accompanied by other congenital abnormalities. In this case report we demonstrate the importance of clinical awareness and of general medical examination when encountering similar patients.

## Consent

Written informed consent was obtained from the patient for publication of this case report and any accompanying images. A copy of the written consent is available for review by the Editor-in-Chief of this journal.

## Competing interests

The authors declare that they have no competing interests.

## Authors' contributions

XZL participated in the surgical procedure and drafted the manuscript. XLC helped in drafting the manuscript and with the critical review of the manuscript. LZ helped in drafting the manuscript. All authors read and approved the final manuscript.
